# Euglycemic diabetic ketoacidosis in a patient with new-onset type 1 diabetes following a ketogenic diet: a potential risk of a dangerous dietary trend

**DOI:** 10.20945/2359-4292-2023-0229

**Published:** 2024-08-05

**Authors:** Burcak Cavnar Helvaci, Beril Turan Erdogan, Didem Ozdemir, Oya Topaloglu, Bekir Cakir

**Affiliations:** 1 Department of Endocrinology and Metabolism Ankara City Hospital Ankara Turkey Department of Endocrinology and Metabolism, Ankara City Hospital, Ankara, Turkey; 2 Department of Endocrinology and Metabolism Ankara Yıldırım Beyazıt University Faculty of Medicine Ankara Turkey Department of Endocrinology and Metabolism, Ankara Yıldırım Beyazıt University Faculty of Medicine, Ankara, Turkey

## Abstract

Euglycemic diabetic ketoacidosis (DKA) is a rare complication of diabetes mellitus (DM) characterized by metabolic acidosis, ketosis, and blood glucose levels < 250 mg/dL. The prevalence of euglycemic DKA is increasing with the popularity of ketogenic (low-carbohydrate) diets. We present herein the case of a patient with newly diagnosed type 1 DM who developed euglycemic DKA following a ketogenic diet. A 22-year-old woman presented to the emergency department with malaise, fatigue, nausea, and vomiting. She had no family history of DM. She had consulted her primary care physician 2 weeks before due to hair loss, numbness, and tingling sensation in her fingertips. Her fasting blood glucose was 205 mg/dL at that time. Reluctant to use medication to control her blood glucose levels, she started a ketogenic diet. On admission, she was conscious, oriented, cooperative, and tachycardic. Her body mass index was 17.6 kg/m^2^. Laboratory tests showed fasting blood glucose of 86 mg/dL, glycated hemoglobin of 10.3%, and elevated insulin levels. Ketone levels in urine and blood were high, indicating ketosis. High anion-gap metabolic acidosis was detected, with a pH of 7.10 and serum bicarbonate level of 12 mEq/L. A diagnosis of new-onset DM and euglycemic DKA was established. She was treated with a modified DKA protocol that included intravenous dextrose-containing serum as fluid therapy, and intravenous insulin infusion was delayed until blood glucose levels increased above 250 mg/dL. The development of euglycemic DKA in our patient was attributed to severe carbohydrate restriction. This case underscores the importance of considering dietary risk factors, particularly ketogenic diets, in the management of DM.

## INTRODUCTION

Diabetic ketoacidosis (DKA) is an acute, severe, and potentially life-threatening complication of diabetes mellitus (DM). It arises from a reduction in circulating insulin coupled with an elevation in circulating counterregulatory hormones. The characteristic triad of DKA includes metabolic acidosis (arterial pH < 7.30 and serum bicarbonate < 18 mEq/L), hyperglycemia (blood glucose level > 250 mg/dL), and ketosis. Rarely, some patients may have blood glucose levels below 250 mg/dL at admission, a condition known as euglycemic DKA (1). The first publication of this condition was in 1973 by Munro and cols., followed by a more extensive case series in 1993 (2). Conditions such as recent use of insulin, gastroparesis, decreased caloric intake, anorexia, excessive alcohol consumption, chronic liver disease, use of sodium-glucose cotransporter-2 inhibitors (SGLT^2^is), and glycogen storage disorders may be risk factors for euglycemic DKA. Other potential risk factors include DKA associated with pregnancy, pancreatitis, cirrhosis, or the use of an insulin pump.

The popularity of ketogenic (low-carbohydrate) diets has been increasing in recent years. These diets are popular in diet books and social media due to their weight loss effect. According to the American Diabetes Association, a low-carbohydrate diet is characterized by a daily carbohydrate content below 130 grams or constituting less than 26% of the total energy intake. External carbohydrates cause fluctuations in blood glucose in patients with type 1 diabetes mellitus (DM); in this context, a ketogenic diet can reduce episodes of hypoglycemia and hyperglycemia along with insulin requirements (3). Patients with type 1 DM are advised to include carbohydrates as 45%-60% of their total energy requirements (4). Notably, patients with type 1 DM following a ketogenic diet may develop ketoacidosis while maintaining blood glucose levels within the normal range or slightly elevated.

We report herein the case of a patient with newly diagnosed type 1 DM who developed euglycemic DKA while following a ketogenic diet.

## CASE REPORT

A 22-year-old woman presented to our emergency department with malaise, fatigue, nausea, and vomiting. She had consulted her primary care physician 2 weeks before due to hair loss, numbness, and tingling sensation in her fingertips. Her fasting blood glucose was 205 mg/dL at that time, prompting her referral to an endocrinologist. She wanted to control her blood glucose levels but was afraid of using medications for that, so she started a ketogenic diet without consulting a specialist.

During evaluation at the emergency department, the patient denied prior weight loss. Her general condition was good. She was conscious, oriented, and cooperative. Her vital signs were normal except for tachycardia, and her physical examination was unremarkable. She weighed 47 kg and measured 163 cm in height (body mass index 17.6 kg/m^2^). She had received a diagnosis of polycystic ovary syndrome 2 years before and did not use any medication for this condition. She denied polyuria or polydipsia. She had no family history of DM. Her blood test results were as follows: fasting blood glucose 86 mg/dL, glycated hemoglobin 10.3%, insulin 2.4 mU/L (normal range [NR] 3-25 mU/L), and fasting C peptide 0.42 µg/L (NR 0.81-3.85 µg/L). Ketone levels in urine and blood were high, and high anion-gap metabolic acidosis was detected. The patient’s lactate level was below 1.0 mmol/L, arterial pH was 7.10, and serum bicarbonate level was 12 mEq/L. Her glomerular filtration rate was 128 mL/min/1.73 m^2^, which was consistent with hyperfiltration early in DM. Liver enzymes were within normal limits. Blood alcohol level was undetectable, and blood salicylate level was normal (<3 mg/dL). Pregnancy, hypothyroidism, and adrenal insufficiency were excluded, and no underlying infectious disease was detected.

Based on the findings, a diagnosis of new-onset DM and euglycemic DKA was established. The usual DKA protocol was modified for the patient; thus, a new treatment protocol was implemented. Dextrose-containing serum was started for intravenous (IV) fluid administration, and IV insulin infusion was delayed until blood glucose level increased above 250 mg/dL. The insulin infusion was started at half the usual weight-based dose for DKA treatment (*i.e.*, 0.05 units/kg/hour). The patient’s treatment was adjusted during follow-up according to blood and capillary glucose, blood gases, and capillary ketones. The insulin infusion and fluid therapy were titrated to maintain blood glucose levels at 200-250 mg/dL. After blood pH was > 7.35 and anion gap was >18 mmol/L, intensive insulin therapy was started, and IV treatments were gradually interrupted. The high anion-gap metabolic acidosis resolved after about 36 hours of treatment. Levels of anti-glutamic acid decarboxylase antibody, anti-insulin antibody, and islet cell antibodies were, respectively, 6.4 U/mL (NR < 17 U/mL), 3.76 U/mL (NR 0-8.2 U/mL), and 69.37 U/mL (NR < 28 U/mL). Her diet was extensively reviewed. Liver diseases and glycogen storage diseases were investigated as possible etiologies. Levels of ammonia, organic acids, amino acids, and long-chain fatty acids in plasma and amino acids in spot urine were normal. No liver pathology was observed on an ultrasonography examination requested to investigate glycogen storage diseases.

After excluding other etiologies, the patient was diagnosed with euglycemic DKA due to severe carbohydrate restriction.

## DISCUSSION

As an acute, life-threatening DM complication, DKA should be treated aggressively. Common DKA triggers include infection, trauma, surgery, and other metabolically stressful events. However, DKA may also be triggered by the omission of insulin doses or an imbalance in the dynamics of physiological insulin requirements and amount of insulin administered. Starvation ketosis can occur even in healthy individuals (*i.e.*, those without DM) (5). This effect is the purpose of the ketogenic diet: it causes the body to metabolize fat energy stores, leading to weight loss due to minimal carbohydrate intake. In individuals without DM, starvation ketosis causes mild metabolic acidosis due to control mechanisms that prevent the overt accumulation of ketone bodies. These mechanisms include increased utilization of ketone bodies in the central nervous system and peripheral tissues and a balance between insulin secretion and counterregulatory hormones. The net effect is a hepatic ketone production rate roughly equivalent to that of ketone consumption. According to McGaugh and Barthel, ketogenic diets lead to short-term weight loss, but most data indicate that their long-term effectiveness is comparable to that of other hypocaloric diets (6). The authors noted that this effect may be due to the challenges of sticking to such a restrictive eating pattern for a long time. They also examined the impact of a ketogenic diet on DM and cardiovascular diseases. For patients with type 2 DM, weight loss achieved with a ketogenic diet is likely to result in short-term lowering of glycated hemoglobin. The authors recommended attention and rearrangement of the ongoing medical treatment to reduce the risk of hypoglycemia (7).

Although health problems and side effects related to ketogenic diets are relatively rare in healthy individuals, they are especially risky for patients with treatment-naïve type 1 DM or ketosis-prone type 2 DM, as it may increase their risk of developing euglycemic DKA (8).

Euglycemic DKA is an uncommon but acute life-threatening metabolic complication of DM characterized by ketoacidosis with low (<250 mg/dL) blood glucose levels. The pathophysiology of euglycemic DKA includes a milder degree of insulin deficiency or resistance and an increased glucagon-to-insulin ratio with carbohydrate deficiency. The risk of euglycemic DKA may be increased in cases with insulin resistance, such as polycystic ovary syndrome and leanness, as in our patient, although this hypothesis lacks enough evidence. The absence of hyperglycemia may delay the patients’ diagnosis and treatment, leading to worse outcomes. Thus, establishing a diagnosis of euglycemic DKA is challenging and requires increased attention.

Euglycemic DKA has received more attention recently due to the increased use of SGLT2is. The risk of euglycemic DKA with SGLT2is has prompted the US Food and Drug Administration and the European Medicines Agency to issue warnings on predisposing factors regarding this complication in patients using SGLT2is (9). Notably, SGLT2is lead to carbohydrate deficit due to glucosuria, resulting in an increased glucagon-to-insulin ratio and metabolic shift from glucose to lipid utilization, resulting in ketogenesis and increased risk of euglycemic DKA. This complication may also be triggered by additional risk factors. This class of medications is relatively new, and our understanding of them is expanding continuously. Results from meta-analyses have indicated a need to prevent euglycemic DKA associated with the use of SGLT2is. Patients with DM and risk factors should be informed about using “sick day rules”; they should be advised to continue taking insulin, measuring capillary glucose levels every 4 hours, increasing drinking of calorie-free fluids, and attempting to follow a usual diet. They should also be cautioned to avoid a low-carbohydrate diet and alcohol consumption and discontinue SGLT2is 3 days before surgery. A multidisciplinary approach is beneficial for patients with euglycemic DKA secondary to SGLT2i use, and this medication should be withheld before and after the euglycemic DKA episode (9,10).

New-onset type 1 DM in adults (*i.e.*, latent autoimmune diabetes of adults) is often mistaken for ketosis-prone type 2 DM. Thus, starting SGLT2i treatment in these patients prepares the ground for euglycemic DKA. The identification of autoantibodies confirming type 1 DM, as in our case, can help distinguish between diagnoses. A case of low-carbohydrate diet-induced euglycemic DKA was first reported in 2015 by Hayami and cols. in a 32-year-old diabetic woman with Prader-Willi syndrome using an SGLT2i (11). Since then, many case reports of severe ketoacidosis caused by administering an SGLT2i during a low-carbohydrate diet in DM patients have been published (12-14). Euglycemic DKA caused by ketogenic diet alone has been rarely reported (15). Yaron and cols. reported euglycemic DKA during pregnancy in a 35-year-old woman with type 1 DM and long-lasting bulimia nervosa who was following an intense and prolonged carbohydrate restriction. She was managed with continuous IV insulin infusion, fluid, and electrolyte repletion (16). Shaikh and cols. published the case of a 28-year-old man with type 1 DM who developed abdominal pain, fatigue, and dizziness 1 week after starting a ketogenic diet; the patient was diagnosed with euglycemic DKA and managed with aggressive hydration and electrolyte repletion (17). Bas and cols. reported the first case of euglycemic DKA in a patient with new-onset type 1 DM due to prolonged fasting during Ramadan (18). Notably, ours is the first case report of a patient newly diagnosed with type 1 DM presenting with euglycemic DKA due to a ketogenic diet. In our patient, a strict ketogenic diet led to inadequate use of insulin, resulting in a masked disease process due to euglycemia.

Some points must be considered during the treatment of euglycemic DKA. First, studies have shown that euglycemic DKA generally takes twice as long to resolve than classic DKA (92 hours versus 35 hours, respectively) (18,19). The underlying etiology of this prolonged acidosis is unclear. Aligned with this, the euglycemic DKA in our patient resolved within 36 hours. The main difference in therapy for euglycemic DKA versus classic DKA is the type of IV fluids and insulin dose administered. Consequently, we started treatment with fluids containing dextrose to prevent hypoglycemia while offering this substrate for use by the tissues when insulin was administered. The devised protocol was effective and safe in preventing hypoglycemia and providing normal glucose levels. Notably, low to normal serum potassium levels may be present in this situation.

In conclusion, our patient had euglycemic DKA triggered by a strict ketogenic diet. This case is remarkable because our patient had new-onset type 1 DM, and euglycemic DKA had developed due to an inappropriate dietary program. In patients with DM and normal glucose levels, euglycemic DKA may be overlooked and treated inadequately. In patients newly diagnosed with type 1 DM, recognizing this condition may be even more challenging. In these cases, assessing the presence of acidosis and ketones will guide the diagnosis. The present case highlights key considerations in the diagnosis of DKA and emphasizes the importance of considering dietary risk factors in the comprehensive management of DM from diagnosis to treatment.
